# Climate change mitigation as a co-benefit of regenerative ranching: insights from Australia and the United States

**DOI:** 10.1098/rsfs.2020.0027

**Published:** 2020-08-14

**Authors:** Hannah Gosnell, Susan Charnley, Paige Stanley

**Affiliations:** 1 College of Earth, Ocean and Atmospheric Sciences, Oregon State University, Corvallis, OR, USA; 2 USDA Forest Service, Pacific Northwest Research Station, Portland, OR, USA; 3 Department of Environmental Science, Policy, and Management, University of California, Berkeley, CA, USA

**Keywords:** regenerative agriculture, holistic planned grazing, social–ecological systems, soil carbon sequestration, resilience, natural climate solutions

## Abstract

‘Managed grazing’ is gaining attention for its potential to contribute to climate change mitigation by reducing bare ground and promoting perennialization, thereby enhancing soil carbon sequestration (SCS). Understanding why ranchers adopt managed grazing is key to developing the right incentives. In this paper, we explore principles and practices associated with the larger enterprise of ‘regenerative ranching’ (RR), which includes managed grazing but infuses the practice with holistic decision-making. We argue that this broader approach is appealing due to a suite of ecological, economic and social benefits, making climate change mitigation an afterthought, or ‘co-benefit’. RR is challenging, however, because it requires a deep understanding of ecological processes along with a set of skills related to monitoring and moving livestock and feeding the soil microbiome. We review the literature regarding links between RR and SCS, then present results of qualitative research focused on motivators, enablers and constraints associated with RR, drawing on interviews with 52 practitioners in New South Wales, Australia and the western United States. Our analysis is guided by a conceptual model of the social–ecological system associated with RR that identifies determinants of regenerative potential. We discuss implications for rancher engagement and conclude with a consideration of leverage points for global scalability.

They're not really about carbon farming, even though that's an outcome… They have a focus on rebuilding resilience into the landscape and with that comes productivity (AUS 5).

## 1. Introduction

The past few years have seen growing interest in the potential for natural climate solutions, including managed grazing, to mitigate climate change (CC) through biological soil carbon sequestration (SCS). Grazing by ruminant livestock takes place on approximately one-third of Earth's ice-free terrestrial surface, comprised of native grasslands, shrublands, savannahs, rangelands, pasturelands and pasture-sown croplands [1–3]. Livestock production on these lands is a significant contributor to rural livelihoods globally [4,5] but has also been identified as a significant source of greenhouse gas (GHG) emissions and land-based degradation due to industrial feed demand and soil erosion associated with overgrazing [6]. There is growing recognition among scientists, however, that land-based GHG emissions associated with rangelands are primarily due to poor management resulting in bare ground and soil erosion [7–9] and that managing grasslands strategically can, in fact, contribute to carbon dioxide (CO_2_) removal from the atmosphere by enhancing SCS [10–13]. In layman's terms, ‘It's not the cow, it's the how’.^1^ The IPCC's [14] Special Report on Climate Change and Land, for example, highlights ‘options with large potential for mitigation in livestock systems includ[ing] better grazing land management, with increased net primary production and soil carbon stocks’. Outside of the academy, there is growing curiosity and enthusiasm about ranchers, graziers and pastoralists, often seen as the problem, becoming part of the solution [15,16]. Paul Hawken's popular book and movement, *Project Drawdown*, for example, lists ‘managed grazing’ as number 16 of the top 100 solutions for limiting global warming to 1.5°C by 2050, predicting that 26 gigatons of CO_2_ could be successfully sequestered by 2050 if managed grazing grows from its current extent of 71.6–749 million hectares over the next 30 years [17].

Little is known, however, about what motivates rangeland managers to adopt the set of practices associated with CO_2_ removal through SCS, variously called managed grazing, cell grazing, holistic planned grazing, adaptive multi-paddock grazing, carbon farming, climate smart agriculture, regenerative agriculture and regenerative ranching. The scholarly literature on the topic is sparse and tends to fall into two categories: (i) quantitative, biogeophysical studies that focus explicitly on whether specific rangeland management practices can meaningfully enhance natural carbon sinks [3,9,18] and (ii) social science studies (both quantitative and qualitative) that focus on ranchers' and farmers’ experiences adapting to CC using ‘climate smart’ management practices, many of which happen to overlap with those thought to sequester soil carbon [19,20]. Thus, while there is a growing trend in the policy world towards integrated ‘nature-based’ approaches to addressing the climate crisis that look for synergies between mitigation and adaptation, academic research on land-based approaches to CC mitigation and CC adaptation typically remains siloed.

**Figure 1.**
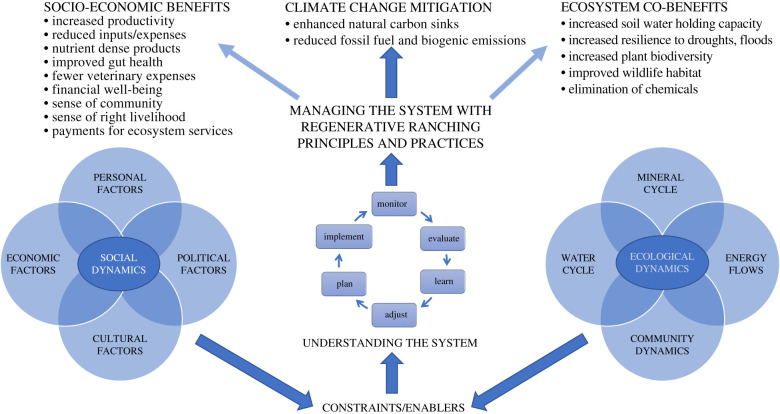
Conceptual model of the social–ecological system in which manager–soil relations occur. Constraints and enablers in social and ecological spheres influence the rancher's ability to understand the system and manage it using regenerative ranching principles and practices. These practices mitigate climate change but, importantly, result in numerous other ecological and socio-economic benefits that are appealing to the rancher. Regular monitoring supports tight feedbacks between the ecological and social sub-systems.

We argue that any effort to engage ranchers in CC mitigation will need to approach research, education and outreach with a more integrated approach that recognizes that rancher–soil relations occur within a social–ecological system (SES) that includes social and ecological enablers and constraints (figure 1) and that mitigation, adaptation and overall system resilience can be integrally related. The best example of this integration that we know of occurs through ‘regenerative ranching’ (RR), a form of regenerative agriculture which, as Gosnell *et al*. [21] explain

goes above and beyond ‘climate smart’ agriculture in that its focus is on enhancing and restoring holistic, regenerative, resilient systems supported by functional ecosystem processes and healthy, organic soils capable of producing a full suite of ecosystem services, among them soil carbon sequestration and improved soil water retention. As such, climate change mitigation and adaptation are incidental to a larger enterprise that employs a systems approach to managing landscapes and communities.

RR is generally associated with holistic management, holistic decision-making, and holistic planned grazing and, as our results suggest, these aspects are what make managing in a way that promotes SCS attractive and sustainable for ranchers [21,22]. RR is challenging, however, because it requires a deep understanding of ecological processes along with a set of skills related to monitoring and moving livestock and feeding the soil microbiome. Additionally, practitioners must navigate a variety of social constraints [21,23].

Recognizing that some degree of SCS and CC mitigation can happen as a result of managed grazing alone, we argue that focusing on the benefits of the broader enterprise of RR is important because some ranchers do not believe in CC, and those who do face more seemingly imperative stressors like debt [24,25]. There are few explicit financial incentives to adopt managed grazing solely because of mitigation, and there are a number of challenges associated with participating in carbon markets [26,27]. Further, many ranchers are extremely risk averse [24,28] and only drawn to adopting practices that they are convinced will reduce their risk to stressors (increase adaptive capacity). Therefore, proponents of engaging ranchers in practices that sequester soil carbon should prioritize understanding what facilitates a shift in their thinking about ranching for resilience, since ‘the climate-smart practices will logically follow, rather than trying to merely incentivize new practices with rational arguments about CC mitigation and adaptation, or payments for enhanced soil carbon sequestration associated with carbon markets, which may have more limited or temporary success’ [21]. The core question about the links between grazing and CC mitigation, therefore, is not just about ecological outcomes related to soil carbon; it is about how a social–ecological regime involving the broader enterprise of RR ‘can help bring about necessary shifts in the systems, structures, assumptions and worldviews in order to support a sustainable society’ [23].

This paper reviews the best available science on links between livestock grazing and SCS, then summarizes results of research on motivations, enablers and constraints associated with ranchers' adoption of RR. Our findings suggest that CC mitigation is not a driving force behind ranchers’ adoption of these management practices; rather, primary motivations occur in ecological, socio-economic and psychological realms. While social enablers and constraints depicted on the left side of figure 1 (e.g. peer pressure, financial constraints, policies and communities of practice) are critically important to SCS outcomes and have been discussed in previous publications [21,23], and ecological constraints influencing SCS have similarly been discussed elsewhere, we focus this analysis on enablers and constraints associated with *understanding and managing the system*, which takes place where the social meets the ecological, as depicted in figure 1. Our discussion is focused on SES dynamics and leverage points that could enhance the global scalability of RR, based on these findings.

## 2. Literature review: linking regenerative ranching and climate change mitigation

There is a growing body of the literature on the connection between rangeland management practices and CC mitigation through GHG emission reductions and enhancement of natural carbon sinks, and on RR in particular. RR aims to enhance soil health and agricultural productivity while meeting each pillar in the ‘triple bottom line’ of sustainability: economic, environmental and social [29]. RR can be ‘scaled up’ to produce rangelands with greater resilience to environmental and economic shocks that often characterize non-equilibrium landscapes [29–31]. Most RR practices leverage ecosystem processes to increase soil organic matter and above- and below-ground biodiversity rather than relying on chemical inputs. RR practices may increase SCS, acting as the driver of other ecosystem services (forage productivity, water holding capacity and biodiversity), though increased plant production and biodiversity also feed back to drive SCS [32].

To achieve these outcomes, regenerative ranchers employ a number of management practices that aim to improve, or regenerate, soil properties such as biodiversity, moisture retention, fertility and carbon content [10,29,33–35]. Practices that are thought to sequester soil carbon include holistic planned grazing (HPG) and ‘feeding the microbiome’ with compost, compost teas and manure, and pasture cropping [15,36,37] (table 1). RR can also include practices that decrease GHG emissions associated with ranching operations, e.g. by reducing the use of chemical inputs, adopting solar-powered water pumps and minimizing machinery use. Additionally, there is increasing evidence that RR can reduce enteric methane emissions, which are often high in grazing systems [13,59]. We do not discuss these latter practices here, nor do we review the literature on reducing the ranch GHG footprint. Instead, we focus on the literature associated with principles and practices thought to contribute directly to SCS.

**Table 1.** Summary of regenerative ranching principles, practices and outcomes relevant to climate change mitigation. Key citations are included.

principle	practice	mitigation contribution	references
reduce GHG footprint	reduce/eliminate use of fossil fuel-derived inputs (e.g. synthetic fertilizers, herbicides and pesticides)	↓ N_2_O and CO_2_	[14,38,39]
	use renewable energy sources, e.g. solar-powered water pumps, etc.	↓ CO_2_	
	reduce use of farm machinery	↓ CO_2_	[40]
	Reduce need for off-farm feed inputs (i.e. hay) by increasing forage productivity and matching stocking rates to forage availability	↓ N_2_O, CO_2_, CH_4_	[10,41–43]
enhance natural carbon sinks	holistic planned grazing	high stock density, short-duration grazing changes ruminant behaviour to promote more uniform (versus patchy) grazing, preventing overgrazing. This reduces bare ground and soil erosion, and increases the competitiveness of perennial species, which have deeper/larger root systems, crucial to SCS and water retention	[8,10,11,13,44–53]
	soil amendments, e.g. biochar, compost and compost tea, manure	promotes SCS by alleviating nutrient limitations and increasing plant productivity and microbial biomass	[54–56]
	pasture cropping	converting cropping systems to pasture cropping, or intercropping cereals into established pastures can increase SCS via perennialization	[37,57,58]

Undergirding the suite of RR practices highlighted in table 1 is a mindset characterized by systems thinking and adaptive management [60], which is a core element to success on rangelands [61]. Regenerative ranchers use adaptive management informed not only by iterative ecological experimentation but also through tying their on-ranch decisions to long-term financial, social and market goals [22,28,62]. The ability of regenerative ranchers to make adaptive grazing decisions based on short- and long-term stressors such as seasonal forage availability, fire, drought, access to markets and labour inputs increases their likelihood of success under dynamic rangeland conditions [50]. The use of adaptive grazing management by regenerative ranchers to meet their ecological goals (i.e. reduced bare ground and increased perennialization) indirectly promotes rangeland SCS via appropriate responses to changing conditions [10].

### 2.1. Holistic planned grazing

HPG is a management approach that manages for plant recovery periods using high-intensity, short-duration grazing events with a principled focus on the appropriate timing of pasture use, leaving adequate plant biomass, rest and recovery periods, and adaptivity. This approach is also referred to in the literature as adaptive multi-paddock grazing (AMP) [63] and management intensive grazing (MIG) [64]. Cell and mob grazing are also short-duration, high-intensity management styles, but do not emphasize adaptivity or social–ecological feedback loops, and are generally more prescriptive. We acknowledge that HPG, AMP and MIG all refer to the same grazing management principles, but use the term HPG owing to its more explicit connections with the holistic decision-making frameworks used by the majority of our interviewees.

A number of studies have found that HPG can positively affect rangeland soils. These outcomes include (i) increasing soil respiration, topsoil depth and soil organic matter [10,45,46,48,65]; (ii) improving water holding capacity and associated hydrological functions [10,44,46,66]; (iii) increasing the retention and availability of soil nutrients [10]; and (iv) reducing bare ground and stimulating vegetation growth [10,44]. By optimizing soil conditions, some researchers assert that HPG also facilitates increased SCS, contributing to CC mitigation [13,47,67,68]. Others disagree, arguing that rangelands are weak carbon sinks, that grazing practices have little effect on SCS [69] and that any positive impacts of grazing on SCS may be offset by livestock methane emissions [70,71]. New work [23], however, suggests that this disagreement may be more a result of terminology differences than a substantial difference of opinion. Scientific evidence on the impacts of HPG on SCS remains limited (and therefore controversial) by challenges to conducting research on the topic including the following:

1. Small-scale and short-duration experiments, the norm, do not represent on-ranch conditions [72].
2. Ranchers who practice HPG use a range of stocking densities, rest-rotation schedules and grazing records, making consistent quantitative research difficult.
3. Many grazing studies confound stocking rate with stocking density and grazing treatment.
4. Controlled experimental grazing treatments are untenable to ‘adaptability’, a key component of HPG.
5. Heterogeneous rangeland ecosystems exacerbate the already complex conditions of HPG research [73,74]. This high baseline variability can require prohibitively high sampling intensities for adequate statistical power [1,75].

Below we focus on the driving principles of HPG and its *known* impacts on SCS.

*Frequent animal movement combined with higher stocking densities limits plant selectivity among ruminants, preventing chronic defoliation that can ultimately lead to bare ground and soil erosion*. Moving livestock frequently allows ranchers to follow available forage as it shifts during the growing season. A critical aspect of HPG is ‘to match forage demand to forage availability’ [76], which has a cascade of positive impacts [34]. To meet this objective, land managers adapt both stocking densities and plant recovery periods according to changing forage availability, climatic conditions, exogenous shocks and stressors, as well as to meet social and market needs [10,76]. This is particularly important in arid, non-equilibrium rangeland settings where conditions vary drastically from year to year [30,77].

Shorter, higher intensity grazing events, compared with chronic, continuous defoliation more uniformly distribute grazing pressure across paddocks, which prevents repeated defoliation [78]. This dynamic can prevent bare ground caused by patchy overgrazing, improve pasture utilization and increase perennialization (the transition away from annual plants to perennials)—leading to improved capacity for soils to sequester carbon [44,79–81]. Leaving sufficient plant biomass and litter cover also improves rangeland productivity by increasing root biomass and reducing soil erosion which, in turn, increase SCS, nutrient and water retention, and above- and below-ground biodiversity [10,42,82]. Minimizing bare ground not only maintains existing carbon in the soil by preventing erosion but also increases carbon inputs to the soil via increased above-ground (i.e. plant litter) and below-ground (i.e. root litter) biomass.

In addition to increasing pasture-scale vegetative biomass left after a grazing event, regenerative ranchers often emphasize leaving enough photosynthetic leaf area on *individual* plants by rotating animals before they are able to take a ‘second bite’. Leaving plants with enough photosynthetic leaf area (a) ensures that they are able to regrow following a grazing event and (b) maintains them in a vegetative (younger) versus reproductive (more mature) state for a longer period of time [83,84], which more rapidly assimilates carbon in roots and shoots [8,85]. This same dynamic can reduce enteric methane in grazing animals, as vegetative plants are less fibrous and higher in energy [86].

*Optimizing pasture rest and recovery between grazing events is an important driver of positive SCS outcomes in HPG* [87,88]. Studies point to even more pronounced benefits of rest in arid and semi-arid rangelands, which are often limited by sporadic precipitation events and low net primary productivity [89]. In a recent analysis of grazing management and SCS, Sanderman *et al*. [90] concluded that rest periods providing adequate plant recovery were the most significant predictor of increased SCS in grazing systems. Varying rest and recovery periods across rangelands with HPG also initiates and accelerates the process of perennialization [52]. In RR circles, there is a shared appreciation for native perennial grasses because of their longer lifespans and extensive root systems, which are important in both sequestering and stabilizing below-ground carbon [51]. Recent work indicates that HPG has increased perennialization, even in the rangelands of California dominated by annual plants [52,53]. In addition, some regenerative ranchers also choose to explicitly seed perennials into their pastures. However, one major challenge associated with transitioning to perennials is tolerating weeds for a period of time while processes of succession take place [91].

### 2.2. Feeding the microbiome: soil amendments and pasture cropping

Some regenerative ranchers amend soils by ‘feeding the microbiome’ using biochar, compost and compost teas to increase SCS. For example, results from the Marin Carbon Project have shown successful increases in SCS following a single compost application in rangelands [54]. Similar boosts in SCS have been observed from manure [92,93] and biochar [55]. However, these practices can be costly, and benefits must be weighed against potential increases in soil GHG fluxes of nitrous oxide (N_2_O) and methane [94].

Another strategy used by regenerative ranchers to feed the soil microbiome and support perennialization and, by extension, SCS, is pasture cropping. Pasture cropping involves either shifting cropping systems to include a pasture rotation, or seeding cereal crops into established pastures during the dormant season [57]. The introduction of perennial pasture phases into cropping systems has significant SCS benefits. This form of pasture cropping simultaneously reduces tillage and bare ground while also increasing root biomass [58]. Although sowing winter cereals into perennial pastures has not been shown to significantly increase SCS, recent studies show increased financial profitability from diversifying revenue streams while maintaining baseline perennial soil carbon stocks [58,95,96]. Peer-reviewed literature on the SCS outcomes of pasture cropping is limited, though our interview data suggest that this practice is gaining popularity among regenerative ranchers, especially in Australia.

### 2.3. Summary

While there is a growing body of literature on RR, few studies have directly evaluated its impacts on SCS and potential CC mitigation. The current body of research has catalogued that RR can have positive ecological impacts on forage quality, quantity and composition; reduction in bare ground; water holding capacity; resilience; wildlife habitat; and potentially SCS. However, the challenges in research and sampling design, as well as study longevity, mentioned above, need to be addressed to better understand the long-term SCS potential of RR.

Research regarding RR and its effects on SCS has largely been conducted by biophysical scientists. There is little social science literature that investigates in an in-depth way ranchers' interest in and abilities to engage in RR practices that might help sequester carbon, and what enables or constrains them [21]. Ranchers who want to adopt RR practices may be stymied by social, psychological, economic or regulatory barriers to changing their operations; or by physical barriers on the landscape. Gosnell *et al*. [21] summarize these ‘zones of friction’ which challenge the adoption of RR. To address persistent gaps in understanding of these dynamics, we turn now to interviews conducted with ranchers in the United States and Australia that shed light on what motivates, enables and constrains them in adopting the kinds of RR practices reviewed here, and, as a result, potentially contributing to CC mitigation.

## 3. Methods

Our results draw on data from two projects focused on ‘climate smart’ ranching. The first (2009–2013) was focused on how ranchers could be incentivized to adopt ‘carbon friendly’ land management. One component of this project involved interviews with 23 ranchers in Montana, Wyoming, New Mexico and Colorado who had generated carbon credits through the Chicago Climate Exchange's (CCX)^2^ soil carbon sequestration protocol [26,27]. Of the interviewees, 20 were males and 3 were females, all were white, and all were cattle producers. Most lived on family ranches, although some were managers of larger corporate ranches because the larger the ranch, the more financially feasible it was to participate in carbon markets. A second component of this project included interviews with 21 graziers in the ‘wheat and sheep belt’ of the state of New South Wales, Australia, known for their experience in ‘climate smart’ regenerative agriculture [21]. The vast majority of these graziers self-identified as either regenerative farmers and/or holistic management (HM) practitioners, and some were also HM educators. They produced sheep or cattle; 18 were males, 3 were females, and all were white.

The second project (2017–2019) focused on benefits and challenges of ‘climate smart’ strategies used by eight progressive ranchers in the Blue Mountains of northeast Oregon (6 males and 2 females). All were white with the exception of one Native American. Methods for both projects included semi-structured interviews, participant observation and document analysis. We used CCX records (for the first study) and purposive sampling [97] to identify interviewees. In our presentation of results, we indicate whether interviewees were from the US West and which state (US XX#) or Australia (AUS #). Livestock producers in Australia generally identify as ‘farmers’ or ‘graziers’ rather than ‘ranchers’; we use the term ‘rancher’ to refer to them all here.

The ranching context in the American West and in Australia bears some similarities and some differences. The western United States contains an estimated 426.7 million acres of rangelands, roughly half of which occur on federal lands, and the remainder of which lie primarily on private lands [98]. Consequently, many ranchers in the states where we worked move their livestock seasonally between their private ranches and public lands where they maintain access to specific grazing allotments. Many also produce hay to feed their animals in winter. By contrast, rangelands in New South Wales' Great Dividing Range are largely private. Ownerships are large, and ranchers maintain their livestock on their private lands year round while also engaging in some crop production. Climate conditions in both locations are generally arid or semi-arid and are predicted to become increasingly hot and dry with global climate change [99]. Cattle production across much of Australia and the US West is expected to become increasingly vulnerable to climate change in the future [100].

Interviews and subsequent coding focused on ranchers’ management philosophies; how they came to be interested in RR; constraints and enablers associated with activities that support CC mitigation; and their thoughts on how RR might be scaled up and mainstreamed. Interviews lasted from 1 to 3 hours and were conducted primarily in person, with a few telephone interviews. Interviews were recorded and transcribed and detailed field notes were written following each interview. Analysis of the interviews was conducted using a thematic analysis approach whereby repeated coding, sorting and categorizing were conducted using NVivo qualitative analysis software [101–103]. Coding themes we explored for this paper included motivations, challenges and benefits associated with RR; and levers and barriers of/to change. Results of our coding informed the development of our conceptual model (figure 1) and we selected exemplar quotes to represent these themes in the following section [97,104,105].

## 4. Results: motivations, enablers and constraints associated with regenerative ranching

### 4.1. Motivations that transcend mitigation

The initial focus of the first study (2009–2013) was on understanding how payments for SCS might incentivize ‘carbon-friendly’ ranch management and contribute to CC mitigation. Interviewees in the US, where ranchers participated in the CCX [26,27], and in Australia, where the government was developing a Carbon Farming Initiative (CFI) that would potentially pay sheep and beef farmers for SCS, consistently reported that their interest in RR practices had little or nothing to do with the promise of carbon payments. Ranchers in New Mexico who transitioned from conventional grazing to HPG over a period of 10 years, and then earned US$80 000 in CCX payments, for example, concluded that carbon payments alone would not have justified the financial, emotional and mental commitment their transition involved.

You really have to be committed to follow through with the whole [holistic] grazing thing. It has to be something that you want to do for the health of the land and for the sustainability of your ranch. It can't just be for money. The carbon credit stuff is just like, you know, gravy (US NM1).

A Montana rancher expressed a similar sentiment saying,

We're improving our agricultural system irrespective of the carbon market. We're doing those practices not because we expect to make money from selling carbon, but because that's the way to better sustain ourselves here … by having more cover, capturing more moisture, taking advantage of what we can in an arid environment (US MT1).

Australian participants expressed similarly lukewarm feelings about the prospect of CFI payments being a motivator to do RR. Rather, there are a number of benefits to increasing soil carbon and co-benefits related to other aspects of conservation and production that motivate the rancher. From a productivity and prosperity point of view, according to one farmer, ‘*it's frighteningly logical*’ (AUS 8). Others concurred:

My goal was to build resilience, to have grasses growing all year round, being able to fatten stock all year round and those sorts of things, and to grow green grass in dry periods and all that. It's later on you realize that, for that to happen, you've got to be building your soil, so you've got to be building your soil biology, your soil carbon, which gives you the resilience (AUS 17).

The most commonly mentioned benefit of RR was the increase in ‘deep ground cover’ associated with increased SCS and perennialization, which translated to more forage for livestock and greater resilience to stressors like droughts, floods and even freezing temperatures since ‘*the organic activity keeps [the ground] warm in winter. Your frosts aren't as severe as they would be*’ (AUS 8).

When you go from a lot of bare ground to really, really high quality cover and then it rains, when it does rain out there, instead of overland flow you now have subsurface flow and the water is entering the creeks as it should. The hydrology has been restored and the water is reentering the creeks from the sides and that stretches that greenbelt out … so it's producing riparian vegetation that wasn't there (US WY1).

Also related to resilience, regenerative ranchers' lack of dependence on inputs results in less vulnerability to financial shocks and stressors.

As farmers and price-takers, you've got very little influence over what you get for your livestock, or your produce, or anything you're selling, but full control over your input cost; [thus RR is] very low risk, because you're not spending any money on anything (AUS 8).

Because you're working quite closely with nature your cost base is always going to be lower because you're not fighting so you don't have the chemicals, you don't have some of the animal health things, yeah. So I guess there's competitive advantage (AUS 1).

For example, one Australian farmer (AUS 4) estimated that his costs were 80% lower since transitioning due to eliminating chemical fertilizer and insecticides and reducing fuel costs. Moreover, the only equipment RRs typically use is ‘*very basic one-wire electrified fencing … just to get a psychological barrier instead of a physical barrier … you can do that for fifteen percent of the cost of a conventional fence*’ (AUS 8). The farmer quoted here contrasted his operation to more conventional operations that rely on ‘fortress fencing’ to control their animals.

Because most regenerative ranchers use ‘low stress livestock handling’^3^ to facilitate frequent movement of their animals, they enjoy a different relationship with them which reduces the need for containment and force and also translates to fewer veterinary bills. Further, because HPG has animals constantly on the move, they are not as susceptible to worms, which eliminates the need for ‘drenching’ (the process of administering chemical solutions by mouth to prevent parasites).

We don't spend a lot on animal health with drench, because the cattle are moving around. The worm cycle's broken, so the cattle are in great condition. Haven't been drenched for five or six years for any worms. There's 120 days between grazes, so there's no host animal for the worms outside the cow (AUS 8).

A US rancher (US OR2) observed that since HPG leaves 4–6″ residual grass, and worm eggs are on the bottom 4″ of a grass plant, his cows are less likely to ingest eggs in the first place. Thus, not only does RR have many environmental benefits but it also has financial benefits that serve as motivations for adoption.

Finally, for many, the personal benefits of this way of life are a major driver of adoption and persistence. While the first few years of transitioning require quite a bit of work, the new RR system, according to most, is much easier to run: ‘*Since I've changed here, there is a lot less work to do. It's become so it's really, really, easy. Less work, and make more money. It's really simple stuff!’* (AUS 4). Another farmer referenced legacy considerations: ‘*Less chemicals, cleaner environment. Bring your kids up in a healthy environment, and you can pass it on to the next generation in good economic and ecological condition. You can live a better life*’ (AUS 8).

This catalogue of compelling reasons to adopt RR and contribute to CC mitigation along the way bodes well for its global scalability—even in the absence of a price on carbon. But all of our interviewees agreed that these benefits do not come easily. Along with a steep learning curve, there is a need for a philosophical commitment to this approach to decision-making; and a passion for working closely, thoughtfully, and iteratively with nature, land and animals. As figure 1 suggests, a rancher's ability to enhance natural carbon sinks on the grasslands he/she manages is influenced by enabling and constraining factors that operate in both social and ecological realms and shape manager–soil relations and the regenerative potential of the operation. Below we highlight some of the most frequently mentioned considerations associated with the practice of RR, focusing on the core issues of managing the system and its critical prerequisite, understanding the system, since adopting RR management strategies might not make sense (or even be possible) without that understanding [60]. As stated above, although important enablers and constraints occur in the social realm [21,23] (see table 2 in [21]), space constraints prevent us from addressing all aspects here.

### 4.2. Understanding the system

A key finding from our interviews was the necessity of understanding fundamental ecosystem processes and how the system can be manipulated to mimic natural processes in order to be a successful regenerative rancher. To enhance soil health and natural carbon sinks, ranchers must not only understand the *mineral cycle*, i.e. how carbon cycles between the atmosphere, plants and soil via photosynthesis; but also the *water cycle* and what determines whether rain evaporates and runs off the soil surface or sinks in and recharges groundwater; *energy flows* associated with the conversion of solar energy into grass and ultimately beef; and *ecological community dynamics* involving ecosystem succession, relationships and interdependencies. The more one understands about how nature works, the more potential there is for regeneration, SCS, and, incidentally, CC mitigation. Conversely, an inadequate understanding of the system constrains a manager's effectiveness. For example, a rancher can practice managed grazing, but without the ability to ‘see’ and interpret subtle signals for how the land is responding, he/she will be less able to adjust management to address underlying problems and leverage natural processes.

Not only can we not see an ecosystem, we can't see a water cycle, we can't see a mineral cycle, so we learn to look for symptoms. We see these symptoms that are indicators of how well that particular ecosystem process is working (US OR2).

Ranchers learn about these ecosystem processes and dynamics from teachers, classes, trainings and workshops, many of them associated with HM, as well as peers. Often the learning curve is quite steep, but through experiential education, hands-on planning exercises and visits to demonstration projects, they gain confidence to experiment and try new things. Once people start to understand how the system works, they often wonder why they have been managing conventionally since it no longer makes sense.

A key feature of systems thinking involves shifting one's focus from the end product, beef, to nurturing and fostering the conditions that make the beef possible, namely grass and soil. With this shift, animals and microbes become tools to promote more deep-rooted native perennial grasses and less bare ground. Beef for consumers is, therefore, a by-product of this focus on grass, and the same can be said for carbon. That is, carbon is a means to an end and not an end in itself.

Everyone talks about carbon, but to increase carbon you've got to fix the other problems first. You've got to fix soil structure and all the other things. But preceding that, you've got to go back to plants. Plants are what drives pretty well everything. Like, soil health, soil structure…it's all microbial health. And the carbon, I think, it comes well after all that, the increase in carbon. Plants are the drivers of it. It's all about diversity of species (AUS 4).

When ranchers truly understand how different parts of the system work together, they begin to appreciate the value of complexity and diversity and nature's innate intelligence. They begin to realize that the modern approach to managing a pasture with chemicals and a very few introduced annual species makes little sense, and that, to be resilient, the pasture needs to look and function more like a grassland, populated with a diversity of deep-rooted native perennials.

Seeing the world this way changes the way people view their relationship with nature. ‘*You shift toward acknowledging, recognizing that you are part of the whole and you become more in flow with it rather than being at the pinnacle of the top of the food chain looking down*’ (US OR2). This involves giving up some control since a complex system cannot be controlled, and focusing on the things that *can* be controlled. For example,

You've got no control over actual rainfall, but full control over effective rainfall. If you've got bare ground and you get an inch of rain, most of it is going to run off into the creek, but if you've got full ground cover and all the energy's taken out of the water before it hits the soil surface, it stops the compaction, soaks in, and then the ground cover holds the water there, so we can control that (AUS 8).

With this understanding, ranchers can work with animals and microbes to foster complexity and biodiversity and co-produce the landscape they desire. Although understanding the system is a fundamental enabler of RR, it can also operate as a constraint since systems thinking requires a significant cognitive shift and a new way of seeing the world.

### 4.3. Managing the system

You see, nature really—it doesn't want to have bare ground. It wants to have, at least in this grassland scenario, it wants to be a grassland and it wants to be perennial, but we keep stopping it from doing that. So, having enough confidence to step back and let nature drive it for us is one of the big hurdles (AUS 4).

At the most basic level, the overarching goal of RR is minimizing bare ground: *‘Profit is important, but the landscape is the number one factor. I'm aiming—you've probably heard this, it's a bit of a cliché now—but I'm aiming for a hundred percent ground cover a hundred percent of the time’* (AUS 7). The interviewees discussed insights regarding best strategies for achieving this goal and reflected on what makes it so challenging. Common to all practitioners of holistic decision-making is to ask, before every management decision: *‘Are we simply treating symptoms or are we addressing root cause of the problem? Is this the biggest bang for my buck and time and my money spent?’* (US OR2). This approach permeates all aspects of managing a system holistically and regeneratively. We focus here on two core aspects of implementing HPG to reduce bare ground: figuring out when to move one's animals and when to destock using a variety of monitoring strategies.

#### 4.3.1. Knowing when to move animals

As described in §2, the main tool used by regenerative ranchers to reduce bare ground is HPG, which involves a high intensity, short-duration approach to moving animals in and out of a system of pastures or paddocks. The crux of this approach is figuring out when to move the animals to create the right amount of impact and rest in order to stimulate grass growth (and soil organic matter accumulation).

So, multiple grazings—plants need to rest properly and then graze when it's completely rested. Graze, rest, graze, rest, graze…so all that time, you're basically just pumping carbon into the soil. Yeah. So it's pulsing it into the soil. That's how you get the big amounts in there (AUS 18).

Unlike rotational grazing which adheres to a calendar, HPG involves moving livestock in response to information gained from monitoring the land and animals. Decisions are ‘*determined by the plant growth, constantly monitoring and adjusting… allowing for recovery time at the end*’ (US OR2). As this Oregon rancher explains,

It's about resting your grass as much as anything. People can forget that part. They're really not getting it right, because that cycle of rest is what the soil and the plants need, it's awesome. That's the piece that we incorporate. You can put herds together and really have a great impact. In some pastures, you may only be there once all summer long. It gets hammered, and then it comes back with full vigour. It gets to go to seed, it rests the rest of the season (US OR5).

While plant and soil recovery through rest is essential to regeneration, interviewees emphasized that it must be balanced with animal impact from hoof action, especially in ‘brittle’ environments where decomposition must happen through the gut of grazing animals due to the lack of consistent moisture at the soil surface. According to interviewees, the physical act of trampling standing dead plant biomass facilitates decomposition in these environments by getting the plant material that would otherwise oxidize to the soil surface where the decaying matter can instead cover the soil surface. The animal impact caused by hooves thus breaks up the soil surface improving rainfall effectiveness, buffers it from extreme temperatures and creates habitat for microorganisms that are critical to healthy soils.

Knowing how to balance rest and impact requires being able to read the land and read the animals, looking for indicators related to the ecosystem processes described above. ‘*You have this awareness. You don't just move your cattle, you go look at the ground*’ (US OR3). For many ranchers, this requires new monitoring skills.

It's just constant monitoring. Because you acknowledge you're going to be wrong, and you're going to be wrong most of the time. So you need to keep on monitoring… the earliest possible monitoring. What would we look for? Soil surface, plants not recovering, plant wilt, anything like that (AUS 22).

The condition of plants in the paddock is the main indicator of when to move livestock into a paddock. The rancher is looking for signs that the plant has recovered adequately from the last grazing: ‘*a healthy plant will have some live and some dead material in it, the litter that is dead from last year maybe, maybe it's a little grayer colored*’ (US NM3). Ranchers also use animal-based monitoring, which includes ‘dung scoring’—looking at the size and shape of the dung for clues about the condition of the pasture—and observing animals' behaviour. These data inform decision-making regarding when to move livestock out of a pasture.

First indicator is the size and shape of their dung. That's the first thing I look at when I go into the paddock. That tells me the digestibility of the feed. And so that automatically tells me pasture quality (AUS 3).

Other signs are behavioural or related to body condition: *‘walking fences, hanging on waters, rumen full on the left-hand side, protruding. By the time they are bellowing at the gate, you've missed the boat. Those animals are distressed. They've run out of food or shade or water’* (AUS 22). This rancher described what could be learned by observing individual animals and using visual and auditory cues:

It's a case of being observant. If you watch them, you drop the wire, as soon as they moved in, their heads would go down, and you had all your herd eating. The minute a head popped up, it was time to move them again. And that was literally 5-10 minutes. Or you could just listen to them. The minute they started running short, you'd hear a ‘Moo’ and that was time to move again (AUS 22).

Another rancher claimed he could tell when his animals needed to be moved from 10 km away (flying overhead) by observing the herd's behaviour as a whole (AUS 8).

The HPG practice of working with large herds is, thus, a double-edged sword since both positive and negative impacts can happen quickly. A large herd mishandled or untended can cause tremendous damage:

But the impact on where the animals are is high. So what it means is, you can't just go away and forget it. If you go away and forget it, you'll have a disaster on your hands awfully quickly. Those cattle down there in the paddock for two more days than they should be there, they will turn it into an absolute desert (AUS 5).

#### 4.3.2. Knowing how many animals to graze

One of the most difficult decisions a rancher has to make has to do with destocking since herd size is commonly equated with wealth. Many of our interviewees contrasted their approach, which involves carefully matching herd size to resource availability, to conventional thinking about herd size.

The common practice is to be trying to run a number of animals that, you know, on the books produces a profit. Generally what happens, when the prices of products fall, people increase their impact. So they try to run more to get the amount of income that gives them a profit, and that's completely the opposite to what we would be doing (AUS 5).

The reason destocking proactively is so important to regenerative ranchers is twofold: to avoid overgrazing and to avoid spending money on supplemental feeding, which is considered anathema to most.^4^

If you're feeding hay you've got too many cows. A cow either makes a living off the ground or out of the sack, more or less. If you're having to pour it out of the sack, it's not getting it off the ground (US NM3).

We don't hand-feed … if we haven't got the grass in front of us, we haven't got livestock. So, we have to become very good at growing grass and rationing the grass so that it gets us from one growing season to the next growing season (AUS 7).

A key management strategy for regenerative ranchers is, therefore, to keep close tabs on weather forecasts and regularly calculate ‘how much grass is in front of us'. If a dry period is anticipated, the rancher proactively sells livestock before problems arise: ‘*We sell stock numbers down in the drought instead of letting the cattle bare all the country out, and then you lose all your carbon, all your good work*’ (AUS 8).

This strategy requires a different way of thinking about wealth—as associated with the health of the land instead of the size of the bank account. Regardless, destocking can be emotionally challenging, as this rancher describes:

When we were in a drought … it was really, really tough to realize oh, man we need to cut back our numbers. But, we knew that we had to. I mean, financially it didn't help us, but we knew we had to have that bank of ecological health, grassland health, in order to actually make it through the drought (US OR3).

One constraint associated with early destocking is that there may not be a market at the time, and the animals may not yet be the right weight for whatever market does exist. What this means is that ‘*you have to have your own marketing system set up*’ (AUS 23). Selling when everyone else does, after the drought hits, guarantees a lower price and fewer options; and, by that time, the ground may be degraded. Box 1 includes the story of one rancher who used RR principles to cope with the Millennium Drought of 2001–2009, the worst drought on record in southeast Australia [114].

Box 1. **A story of regenerative approaches to managing herd size and destocking during the 2001–2009 drought in Australia.**

We made a very fundamental decision to totally destock the property in 2004. Not because we had to, but because we could see the writing on the wall. Going into winter, insufficient rainfall, not much chance of growth because of the cold, and the prospect of destroying the soil surface and all the vegetation with it by the end of winter were pretty frightening. So, having done that, it was a very major decision, a pretty full-on decision, and a very emotional decision, selling 550 cows in one hit. It—we were really glad of it, because we maintained ground cover, and I think we jumped out of the drought a lot quicker than we would have done if we'd just waited through and we didn't know when it was going to end, and we'd be substitute feeding and all those sorts of things. So it was a magic thing to happen in many ways, because it set us up for those sorts of decisions forever, and I think this place has actually benefited more from good drought management rather than good management in good seasons (AUS 24).

To summarize our findings, interviewees were motivated to adopt and practice RR because of its many perceived ecological, socio-economic and psychological benefits, with SCS a secondary benefit. Overcoming significant challenges associated with learning how agroecosystems work and learning new tools for monitoring and managing plants and animals is a prerequisite to reaping the benefits of RR.

## 5. Discussion: understanding social-ecological system dynamics to support global scalability

Results from this research, presented here and in other publications [21,26,27], illustrate the ways in which the practice of managing the ecological system on the right side of figure 1 is integrally related to social, economic and cognitive considerations on the left side. Examining the experiences of ranchers who engage in RR using this type of integrated SES lens has the potential to produce new contributions to the rangeland science literature. As Hruska *et al*. [115, p. 266] note, little research has examined rangelands as an SES, and, when it does, ‘too often, only single cross-system influences are emphasized … such as how changes in resource or social policy affect rangeland ecosystems, without following up to see how altered ecological processes feedback to affect the social system’. What is needed, they argue, are applications of the SES framework to analyze ‘how social and ecological components of the system interact in iterative cycles’. Here, we illustrate with our model and our results how regular ecological monitoring and proactive rangeland management support tight feedbacks between the ecological and social sub-systems. Positive feedback from the environment related to decisions at the soil surface, e.g. reduced bare ground and an increase in deep-rooted native perennials, engenders enthusiasm and motivation to persist in the personal sphere, which results in positive feedback to the environment [21]. ‘*Once you start becoming observant, well, you become holistic*’ (AUS 22). This rancher's observation about links between ecological feedback and human well-being illustrates this point:

In order for us, in order to be happy, literally, you need healthy soil. And I can't stress that enough… where the cows are happy, the soil, you can tell the land is happy. That would be the driver, how do I get there (US NM3).

As this quote suggests, these self-amplifying positive feedbacks occur at the regional scale as well, as clusters of regenerative ranchers and their communities of practice influence and challenge social–cultural norms around food production and create consumer demand for healthy soils and regeneratively raised products [116]. At the global scale, widespread adoption of RR practices has the potential to challenge the agro-industrial complex as their positive social, ecological and economic benefits are weighed against carbon-intensive conventional practices.

Whereas the global agro-industrial complex (for livestock in particular) hinges upon the production and use of commoditized industrial feed products and agrochemicals produced en masse and traded long distances, regenerative ranchers prioritize community learning networks, local ecological benefits, and the reduction or elimination of all agro-feed and chemical products. In this way, the shift from global to local, carbon-intensive to carbon-negative, exploitative to regenerative, among regenerative ranchers erodes the pillars upon which the agro-industrial livestock complex rests. Negative feedbacks between the two parts of the system occur when, for example, peer pressure, the absence of a market for regenerative products and scepticism from the scientific community inhibit the manager's motivation to persist, or when environmental shocks and associated economic impacts overwhelm the manager's capacity to adapt.

A growing interest in RR has climate leaders wondering how to incentivize widespread adoption of these practices; many assume that a carbon market that includes SCS protocols will be a major leverage point. A few farmers suggested that society should be willing to pay for the SCS services that domesticated ruminants provide without the expectation that they are raised for food, since that aspect of livestock production has significant carbon implications, especially in remote regions of the world.

I'd like to think that there's more money to be made out of carbon sequestration than livestock management, that the livestock management becomes a tool for the carbon sequestration. That would be the ultimate goal, where you can go, well I own these animals because I need to sequester this many tons of carbon this year, and this is how I plan to do it, and that would be an amazing outcome. And it should, it should be here by now (AUS 14).

We suggest that while putting a price on carbon and developing carbon markets that allow ranchers to participate is a useful starting point for incentivizing reductions in GHG emissions and providing some extra income for regenerative ranchers, scaling up RR as an approach to livestock production will not result from such ‘top-down’ endeavours alone. Rather, RR and the regenerative agriculture movement more generally should be seen as a grassroots emergent phenomenon, in which food systems are being reshaped from the bottom up by increasingly aware ranchers and consumers.

Given the relevance of RR to CC adaptation and mitigation, human well-being and overall system resilience, an integrated approach to research, outreach and education aimed at engaging ranchers in these practices is needed. Such an approach would emphasize the multidimensional benefits of RR and address both the fundamental prerequisite for RR of helping ranchers understand the ecosystem and how it can be manipulated to support regeneration; and the challenges associated with managing to minimize bare ground and gaining proficiency in RR. Outreach and education are necessary because RR involves a steep learning curve and a deep commitment to learning about ecosystem processes and adopting new ways of thinking, and managing animals and the microbiome in new ways including without the use of chemicals.

Findings regarding why ranchers adopt these practices suggest that communication strategies should focus not on carbon *per se*, but on other benefits of RR, with SCS a positive side-effect. One farmer who teaches classes on composting found that ‘*to engage farmers you've got to talk about grass production because to them that's money production. That's what they are interested in and then you've got to take them back to what drives that grass production*’ (AUS 3). In most farmers' minds, improving soil health is a means to an end (grass production), and SCS is a by-product, not an end in itself.

One communication strategy involves fostering and supporting communities of practice and peer-to-peer learning. This may be the most effective leverage point for realizing the global scalability of RR.

They have a lot more faith in what they've been told if they're being told by a fellow farmer. Yeah, and they can go and see it happening locally because there's a tremendous concept, and this happens internationally, that someone will come and speak about a way to farm and they'll be from another area or another country and the local farmers will say, ‘Well, that's fine for you but it doesn't happen here. It's different here. The climate is different or the soil is different.’ And that's a huge barrier to adoption of new innovation. But if someone is doing it locally and they can see that it works then it will spread much faster (AUS 3).

In Australia, for example, many spoke positively of the Soils for Life programme and the need for ‘safe spaces’ to learn about new ways of doing things and to consider, without pressure, that old ways may not be working.

I think it would be just showing examples of people doing it well, and just keep on showing different examples, which is what the Soils for Life program's about. They're not trying to tell people how to do it. All they want to do is show people how they are doing it better (AUS 8).

Elsewhere, organizations like the Savory Institute, Quivira Coalition and Holistic Management International, all based in the US, are major diffusers of innovation related to RR.

Several interviewees also commented that educational institutions in both Australia and the United States need to do a better job teaching students about complexity and resilience in agroecosystems using integrated transdisciplinary approaches:

The universities are far behind in all of this. They're still teaching very conventional, thirty, forty-year-old agriculture. And it's all standard old stuff. Like, for example, they do botany, agronomy, soil and soil microbial health, all this sort of stuff, as separate things. No one's ever encouraged them to overlay it all. Because it's all interrelated! (AUS 4).

Finally, many proponents of RR argue that there is a need for bigger markets for grass-fed and/or grass-finished beef and regeneratively produced wool, the main products of RR, that go beyond niche markets, if RR is to be ‘scaled up’.

So the people in Sydney, bloody New York, Washington DC, wherever it is, they're the people who've got to change it, and the farmers will follow. The consumer's created the Monsantos of the world, do you know what I mean? So how can they change it? The consumer can change it. They're the only people who matter in this game. They can change it (AUS 23).

There are currently at least two certification schemes associated explicitly with ‘regenerative’ practices that aim to differentiate from conventional or even ‘merely’ organic products: the Rodale Institute's Regenerative Organic Certification and the Savory Institute's Ecological Outcome Verification programme. While focusing on an enhanced role for the consumer in scaling up RR has some promise, this approach has its limitations. First, research suggests that ‘sustainability labels currently do not play a major role in consumers' food choices’ [117]. Second, such labels commodify and capitalize on characteristics deemed useful by consumers [118]; but the added income they may generate does not guarantee that the producer will attain the level of system understanding needed for RR. Third, individualization of issues such as climate change, and thus consumer-driven solutions to solve them, ignores the nature and influence of powerful actors at the root of the problem (e.g. the power of the large meatpacking plants) [119]. Finally, labels can be deceptive. RR can be employed at any point in the grazing lifetime of a ruminant, but conventional approaches may also play a role in livestock production. For these reasons, we believe that there is a much stronger case to be made for supply side rather than demand-side shifts. We argue that the real challenge here is not to expand markets for RR beef, but rather how to get the ‘holistic’ thinking of RR into the hearts and minds of ranchers—both in order to shift the beef industry towards regenerative methods, and to holistically mitigate climate change.

We suggest that a more promising approach to scaling up RR will involve government-led peer-to-peer learning programmes, potentially within the US Department of Agriculture's Sustainable Agriculture Research and Education Program or its Natural Resources Conservation Service (although resistance in the rangeland science community will need to be addressed); or private initiatives that exist via grants, e.g. the Quivira Coalition's Land and Water and New Agrarian programmes, or Western Landowner Alliance's Women in Ranching Network which empowers and trains women in RR. We also advocate for a movement towards ‘regenerative’ approaches within existing institutions like Cooperative Extension, 4H and Future Farmers of America [120]. There are many other incremental policy changes to supplement these shifts that are beyond the scope of this paper.

## 6. Conclusion

RR is a low-cost, low-tech natural climate solution that has potential to contribute to CC mitigation by reducing GHG emissions associated with conventional agriculture and enhancing natural carbon sinks through grazing practices thought to contribute to SCS. While generic ‘managed grazing’ has the potential to sequester carbon, it does not, by itself, manifest the features that make people want to do it. The use of holistic decision-making in the implementation of managed grazing amplifies its effects and increases regenerative potential, and, by extension, CC mitigation potential. It is the rewarding feedbacks that come from practising and thinking holistically that make people want to stay on the path.

A growing number of ranchers, graziers and pastoralists around the world are adopting these practices; but as the results presented here suggest, CC mitigation in and of itself is not a primary motivation. Rather, there are a number of environmental, economic, social and personal benefits associated with RR that serve as motivations, and RR practices are helping livestock producers adapt to the effects of CC. As such, our results demonstrate the synergies between CC adaptation and mitigation, and suggest that they are approached in a more integrated manner.

As climate leaders and policymakers ponder how to incentivize widespread adoption of CC mitigation practices such as RR, it is important to recognize the enabling and constraining factors associated with them. In the case of RR, this means helping producers overcome the steep learning curve associated with it and supporting a commitment to a new way of thinking and decision-making—both of which to date have made adoption challenging. We have proposed a conceptual model based on our research findings that illustrate how constraints and enablers in social and ecological spheres of the SES associated with RR influence the regenerative potential of an operation. These factors occur at multiple scales influencing management actions, feedbacks and cross-scale interactions. We have also proposed that, although top-down incentives such as carbon markets may help incentivize RR practices, more important is recognizing RR as a bottom-up movement that calls for *in situ* research involving producers in the co-production of knowledge (versus simulated paddock experiments), outreach and education to facilitate adoption. This is because sustaining practices that mitigate CC may be difficult without the shift in thinking and understanding of ecosystem dynamics associated with RR.

Our results underscore the win–win nature of engaging ranchers in regenerative agriculture since their contributions to CC mitigation are not seen as a burden, but rather something that makes them better off. While many approaches to CC mitigation involve sacrifice and doing without (e.g. driving and flying less, spending money on tree planting and investing in biochar), RR has its own built-in inherent rewards. These rewards are an important leverage point to employ in communications, incentive programmes and educational strategies aimed at ranchers. Ultimately, ranchers will be most likely to adopt new practices if those practices reduce their risk to stressors and increase their adaptive capacity. RR has the potential to do this, thereby reducing GHG emissions, enhancing natural carbon sinks, and increasing adaptation, resilience, prosperity and quality of life.

## Endnotes

1 A frequently heard argument in the regenerative ranching world, e.g. in this TEDx talk by Savory Institute's Bobby Gill: https://youtu.be/jKXgVK0TQ1A.

2 The Chicago Climate Exchange, which operated from 2003 to 2010, was North America's only voluntary, legally binding GHG reduction and carbon trading system for emission sources and offset projects associated with rangelands.

3 Low stress livestock handling (LSLH) is ‘a livestock-centered, behaviourally-correct, psychologically-oriented, ethical and humane method of working livestock which is based on mutual communication and understanding, not coercion’ [106]. Studies have shown that animals raised under LSLH conditions tend to have improved weight gain, temperament, yield and immunity, and higher overall welfare [107–110]. LSLH is especially useful to regenerative ranchers that are herding animals on large, extensive rangeland landscapes without structured fences, allowing them to more effectively mobilize and distribute animals for frequent rotations. Bailey *et al*. [111] found that LSLH was an effective way to herd grazing animals in and out of riparian areas for riparian restoration. In this way, using LSLH can help regenerative ranchers reach their rangeland and soil restoration goals [112,113].

4 RR can take place in many geographies, some of where grazing all year is an impossibility and thus requires supplemental feed.

## Data accessibility

The qualitative data we used in this article were derived from interview transcripts which are protected by confidentiality agreements under Oregon State University's Human Research Protection Program and Institutional Review Board; therefore, the data cannot be made public.
